# Draft Genome Sequence of *Mycobacterium cosmeticum* DSM 44829

**DOI:** 10.1128/genomeA.00315-14

**Published:** 2014-04-10

**Authors:** Olivier Croce, Catherine Robert, Didier Raoult, Michel Drancourt

**Affiliations:** Aix Marseille Université, URMITE, UM 63, UMR_S 1095, UMR 7278, Marseille, France

## Abstract

We announce the draft genome sequence of *Mycobacterium cosmeticum* strain DSM 44829, a nontuberculous species responsible for opportunistic infection. The genome described here is composed of 6,462,090 bp, with a G+C content of 68.24%. It contains 6,281 protein-coding genes and 75 predicted RNA genes.

## GENOME ANNOUNCEMENT

*Mycobacterium cosmeticum* belongs to a poorly defined group of rapidly growing nontuberculous mycobacteria, which are most closely related to *Mycobacterium frederiksbergense*, *Mycobacterium hodleri*, *Mycobacterium diernhoferi*, and *Mycobacterium neoaurum* (1). It is an environmental organism recovered from water, including household potable water (2) and water collected at a nail salon (1), activated sludge from a wastewater treatment plant (3), and monument sandstones (4). Accordingly, *M. cosmeticum* can use the benzene series as the unique source of carbon (3). In medicine, *M. cosmeticum* is an opportunistic pathogen most frequently implicated in cutaneous granulomatous lesions following mesotherapy (1, 5). Further isolates have been obtained from blood samples collected from patients with indwelling catheters and from sputum specimens (6). *M. cosmeticum* has also been implicated as a gastrointestinal tract pathogen (7, 8). The optimal treatment of *M. cosmeticum* infection is not known, but one isolate has recently been shown *in vitro* to be susceptible to amikacin combined with clofazimine (9).

In this study, we sequenced the whole genome of the *M. cosmeticum* DSM 44829 strain in order to help depict its phylogenetic relationship with closely related mycobacteria and unique metabolic capabilities.

Genomic DNA was isolated from the *M. cosmeticum* DSM 44829 strain grown in MGIT Middlebrook broth at 37°C (Becton, Dickinson, Sparks, MD). It was then sequenced using three high-throughput next-generation sequencing (NGS) technologies: Roche 454 (Roche Diagnostics Corporation, Indianapolis, IN) (10), SOLiD version 4 (Life Technologies, Carlsbad, CA), and MiSeq Illumina (Illumina Inc., San Diego, CA). A 3.3-kb paired-end library was loaded on a picotiter plate and sequenced with the Roche-GS FLX Titanium sequencing kit XLR70. The run yielded 102 Mb with 256,437 passed filters and an average length of 397 bp. The barcoded paired-end SOLiD library generated 1,144,665 reads of 50 × 35-bp length. Finally, a paired-end Nextera library sequenced on MiSeq at 2 × 250 bp yielded 2,618,618 reads with an indexing of 22.89% on the flow cell.

The reads from the various sequencing technologies were first assembled separately. The 454 reads were assembled into contigs and scaffolds using Newbler version 2.8 (Roche). The Illumina reads were trimmed using Trimmomatic (11) and then assembled using the SPAdes software (12, 13). Contigs obtained were combined by using SSPACE (14) and Opera software version 1.2 (15), helped by GapFiller software version 1.10 (16). Some manual refinements using CLC Genomics version 6 software (CLC bio, Aarhus, Denmark) and homemade tools improved the genome. It was found that the *M. cosmeticum* draft genome consists of five contigs without gaps, containing 6,462,090 bp and a G+C content of 68.24%.

Noncoding genes and miscellaneous features were predicted using RNAmmer (17), Aragorn (18), Rfam (19), and Pfam (20). Open reading frames were predicted using Prodigal (21), and functional annotation was achieved using BLASTp against the GenBank database (22) and the Clusters of Orthologous Groups (COG) database (23, 24). Using these tools, it was found that the *M. cosmeticum* genome contains ≥75 predicted RNAs, including six rRNAs, 53 tRNAs, one transfer-messenger RNA, and 15 miscellaneous RNAs. A total of 6,281 genes were also identified, representing a coding capacity of 5,995,551 bp (coding percentage, 92.7%). Among these genes, 926 (14.74%) were found to encode putative proteins and 1,033 (16.44%) were assigned as genes for hypothetical proteins. Moreover, 6,211 genes matched at least one sequence in the COG database using BLASTp default parameters.

### Nucleotide sequence accession numbers.

The *M. cosmeticum* strain DSM 44829 genome sequence has been deposited at DDBJ/EMBL/GenBank under accession no. CCBB010000001 to CCBB010000005.
